# Advancements in the Blood–Brain Barrier Penetrating Nanoplatforms for Brain Related Disease Diagnostics and Therapeutic Applications

**DOI:** 10.3390/polym12123055

**Published:** 2020-12-20

**Authors:** Suresh Thangudu, Fong-Yu Cheng, Chia-Hao Su

**Affiliations:** 1 Institute for Translational Research in Biomedicine, Kaohsiung Chang Gung Memorial Hospital, Kaohsiung 833, Taiwan; suresh120689@gmail.com; 2 Department of Chemistry, Chinese Culture University, Taipei 111, Taiwan; 3 Department of Biomedical Imaging and Radiological Sciences, National Yang Ming University, Taipei 112, Taiwan

**Keywords:** nanoparticle, blood brain barrier, brain tumor, Parkinson’s disease, Alzheimer’s disease, stroke

## Abstract

Noninvasive treatments to treat the brain-related disorders have been paying more significant attention and it is an emerging topic. However, overcoming the blood brain barrier (BBB) is a key obstacle to most of the therapeutic drugs to enter into the brain tissue, which significantly results in lower accumulation of therapeutic drugs in the brain. Thus, administering the large quantity/doses of drugs raises more concerns of adverse side effects. Nanoparticle (NP)-mediated drug delivery systems are seen as potential means of enhancing drug transport across the BBB and to targeted brain tissue. These systems offer more accumulation of therapeutic drugs at the tumor site and prolong circulation time in the blood. In this review, we summarize the current knowledge and advancements on various nanoplatforms (NF) and discusses the use of nanoparticles for successful cross of BBB to treat the brain-related disorders such as brain tumors, Alzheimer’s disease, Parkinson’s disease, and stroke.

## 1. Introduction

**Figure 1:**
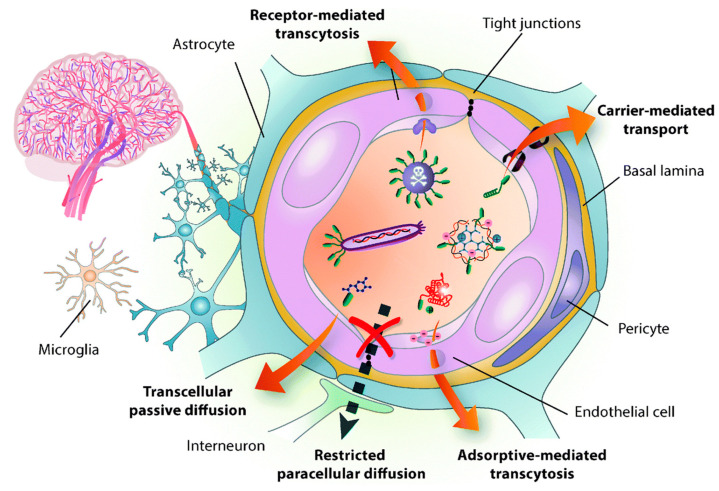
Structure of the blood brain barrier (BBB) and transport pathways across the BBB. Reproduced with permission from Reference [14].

Disorders in the central nervous system (CNS) creates a potential impact on public health and have remained the leading cause of death, mainly in Alzheimer’s disease (AD), Parkinson’s disease (PD), stroke, and brain tumors [1,2,3,4]. However, the current strategies are very far from impressive to treat the CNS, owing to the restriction of BBB for transporting drugs to the brain [5]. As a result, almost 98% of the small-molecule drug and 100% of the macromolecular drugs are unable to enter the brain [6]. The BBB is a physiological structure of the blood vessels in the brain. It not only precisely regulates the entrance and discharge of ions, cells, and molecules between the blood and brain tissue, but it also has an important function in maintaining a microenvironment for reliable neuronal signaling [7]. The BBB is responsible for brain homeostasis and protection and is composed of pericytes (PCs), endothelial cells (ECs), a basement membrane, and astrocytes. ECs form the walls of the vessels through intermolecular tight junctions (TJs). The BBB can restrict the access of molecules into the brain and provides a natural shield against various toxins and infected cells from circulating blood, but it also limits the brain’s uptake of diagnostic and therapeutic agents, thus, reducing therapeutic efficiency [8,9]. An analysis of over 7000 drugs found that only 1% could penetrate the BBB and be active in the central nervous system (CNS) [7,10]. Therefore, the BBB is the main hindrance to noninvasive treatment of brain-related diseases (such as Parkinson’s disease, Alzheimer’s disease, schizophrenia, depression, and brain tumors) because the BBB restricts passage only to necessary substrates from the circulation to the brain tissue [11,12]. The detailed structure of BBB and transport mediated pathways was shown in Figure 1. Other than nutrients, it was shown that small lipophilic molecules (most low-o500Da) are able to cross the BBB effectively and reach the brain [13]. Thereafter, many strategies have been developed to nonspecifically disrupt the BBB and, thus, allow the therapeutic agents to enter into the brain, but these strategies may also allow circulating toxins enter the brain from the blood. Therefore, numerous efforts have been attempted to develop novel strategies, which are able to deliver therapeutic drugs to CNS by crossing the BBB.

In the present review, we mainly focused on advancements in the BBB penetrating nanoplatforms (NFs) for brain-related disease diagnostic and therapeutic applications. The structure and functions of BBB that restrict the brain-targeted therapeutic drugs are described. Furthermore, advancements in the BBB penetrating NFs for treatment of brain tumors, Alzheimer’s disease, Parkinson’s disease, and stroke are discussed. Lastly, future perspectives are discussed to further improve the therapeutic strategies efficiently to conquer the CNS disorders.

## 2. Nanoparticles (NPs) and Their Advantages in Biomedical Applications

In the past two decades, nanomaterials-mediated therapeutic strategies has gained more attention and are widely used in various biomedical applications [15,16,17,18,19]. NPs usually refer to solid colloidal particles at a nanometer scale (1–100 nm). The advantages of using NPs as drug carriers include the following: (i) NPs improve the stability and efficacy of hydrophobic drugs, (ii) NPs improve biodistribution and pharmacokinetics characteristics, resulting in improved accumulation efficacy in the blood and targeted tissues, (iii) adverse effects are reduced due to accumulation at target sites, (iv) and required drug dosage is reduced while increasing therapeutic efficiency. Many studies have been shown that NPs provide different levels of delivery efficiency in various tissues using different strategies [20,21,22]. Importantly, NPs can also be used for various functions in diagnosis, detection, and therapy. For example, magnetic NPs, Au NPs, and quantum dots can, respectively, be used as contrast agents or fluorescent probes for magnetic resonance imaging (MRI), computerized tomography (CT), and optical imaging [23]. Ideally, NPs should be biocompatible, biodegradable, and minimally cytotoxic. Currently, only iron oxide and Au NPs have been approved for use in humans by the U.S. Food and Drug Administration (FDA) [24]. Particularly in brain tumor theranostics, most of the current treatment options were unable to cross the BBB and treat the brain-related disorders. To this end, NPs offer the foremost features in brain tumor treatment including (1) versatile compositions and physical properties, (2) passive targeting of brain tumors, and (3) tunable surface functionality for active targeting. Table 1 represents the key strategies to overcome the current brain-related treatments by nanotechnology.

### NPs Strategies to Overcome the BBB

**Figure 2:**
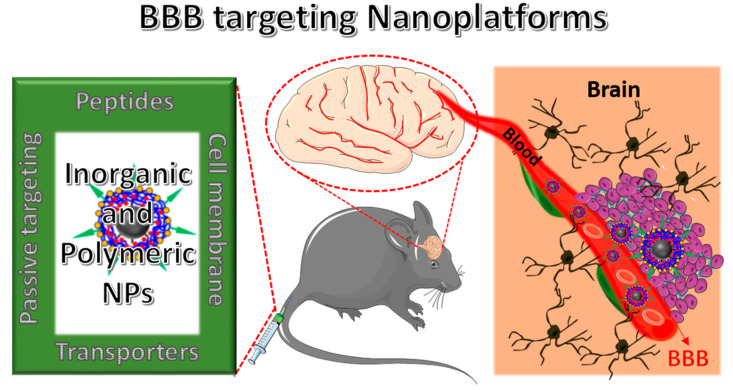
Schematic illustration of the blood brain barrier (BBB)-penetrating nanoplatforms (NFs) for targeted delivery and therapeutics into the brain tissue to treat brain-related disorders.

BBB is the third barrier in the tumor for transferring therapeutic agents [35]. As shown in Figure 1, unlike normal brain capillaries, it is compromised with tight junctions of endothelial cells. The key factor to limit the drug penetration into the brain tumor via the blood stream is mainly due to the high intra tumoral interstitial pressure created by the leaky tumor vasculature nature [36]. Besides, populations of various tumor micro vessels and spatial variability in capillary functions in the tumor microenvironment may also lead to flexibility in penetration [35] As a result, heterogeneous distribution of drug molecules leads to compromise the therapeutic outcome. Although the BBB is intact, it is over-expressed by many receptors and carriers, which can facilitate transport of the specific ligands and cargos (low molecular weight lipid soluble molecules) to the brain efficiently [37,38,39]. The membrane on the BBB is negatively charged, so it exhibits high affinity toward the positively-charged compounds, which could also trigger the internalization and could help to cross the BBB. Remaining molecules require some transport systems to cross the BBB such as carrier-mediated transport (CMT), receptor-mediated transport (RMT), or absorptive-mediated transport (AMT). Firstly, a simple (or passive) diffusion pathway to deliver NPs to brain tumors happens through the “leaky” tumor vasculature, which is often called an enhanced permeability retention (EPR) effect [40]. Therefore, it is strongly recommended that, to achieve good EPR effects, NPs should exhibit their sizes <100 nm in diameter and should be biocompatible to overcome the removal by the cells’ reticuloendothelial system (RES). Second, to further improve the accumulation of NPs in the brain, their surfaces were modified with different kinds of receptors and transporters, which were over-expressed by the BBB. The detailed schematic representation of BBB penetrating NPs’ systems to reach into the brain was shown in Figure 2. By understanding these features, several NPs’ strategies were successfully developed to deliver the therapeutic drugs across the BBB and enhance the accumulation of drugs at a therapeutic site [41,42]. Table 2 summarizes some receptors and transporters, which were over-expressed on the BBB.

Besides the EPR effect of NPs, NPs’ surfaces are modified with receptors and facilitate to deliver the therapeutic drugs and NPs efficiently. For instance, Qiao et al. used lactoferrin-conjugated Fe_3_O_4_ NPs to cross the BBB via receptor-mediated pathways [51]. Georgieva et al. developed G23 peptide-modified polymersomes to penetrate the BBB, and found G23 peptide-modified polymersomes successfully accumulated in the cortex, striatum (forebrain), midbrain, pons, and cerebellum [21]. The peptide G23 was identified by means of phage display with ganglioside GM1 as the target [52,53]. GM1 is a glycosphingolipid ubiquitously present on the endothelial surface. Cheng et al. combined trans-activator of transcription (TAT) peptides, doxorubicin (DOX), and Au NPs to synthesize transactivating transcriptional activator (TAT)-conjugated and doxorubicin (DOX)-encapsulated Au NPs (TAT-DOX/Au NPs) and TAT-DOX/Au NPs to enhance malignant glioma imaging and therapy [54]. In in vivo test results, confocal images showed TAT-DOX/Au NPs accumulated in the brain tumor region. Stojanov et al. prepared prion-targeted and GM1-targeted polymersomes to observe in vivo biodistribution in mice brains because GM1 ganglioside and prion protein serve as potential transcytotic receptors at the BBB [55]. Koffie et al. used poly(n-butylcyanoacrylate) dextran polymers coated with polysorbate 80 (PBCA NPs) to deliver BBB-permeating molecular imaging contrast agents into mice brains for an in vivo MRI [20]. When mice were treated with Hoechst alone, no Hoechst signal was observed in the mouse brain, but a Hoechst signal was observed inside the brains of Hoechst-carried PBCA NP-treated mice, showing that the PBCA NPs crossed the BBB and released the Hoechst into the brain. In addition to BBB-penetrated peptides, Monoclonal antibodies against the transferrin and insulin receptor were also conjugated on the surface of NPs, and these antibody-conjugated NPs could then specifically target OX26 (transferrin receptor) and 83–14 (insulin receptor) present on the blood-facing apical surface of endothelial cells, resulting in NPs being successfully used in animal models to deliver therapeutics across the BBB [56,57]. Although NFs offer good beneficial advantages, restriction of their permeation through the BBB is even more pronounced due to the larger sizes of NPs. Even though utilizing various kinds of delivery methods, e.g., using receptor-mediated strategies, the efficiency of delivering nanoparticles into the brain is insufficient to fully exploit their therapeutic and diagnostic potential. Several studies recently found that the EPR effect is highly heterogeneous both intra-tumorally and inter-tumorally as a result of failing to translate into clinical applications [58]. Therefore, another prominent strategy was developed that is temporarily opening the BBB to enlarge the pore size, which could allow compounds or NPs to directly diffuse into brain [59]. Temporarily opening the BBB could be achieved by several physical and pharmacological methods such as chemical compounds enhanced BBB permeability, receptor-involved changing of tight junctions, and a focused ultrasound. The detailed advancements and therapeutic strategies to treat the CNS disorders are discussed below.

## 3. BBB Penetrating Nanoplatforms (NFs) in Biomedical Applications

By understanding the structure of BBB and utilizing the beneficial advantages of surface modified-NPs, several NFs were successfully applied in various biomedical applications with significant outcomes. Thus, in here, we are more focused on the BBB penetrating NFs, specifically for brain tumor therapy, Alzheimer’s disease, Parkinson’s disease, and stroke applications.

### 3.1. BBB-Penetrating NPs for Brain Tumor Therapy

Malignant gliomas are primary brain tumors derived of glial origin, and 70% of glioma patients survive less than 15 months past diagnosis, even with surgical excision and/or chemo radiation therapy [60,61]. Unfortunately, radio therapy causes serious side effects such as post-radiation leuko-encephalopathy, nerve damage, hair loss, vomiting, infertility, and skin rash. As well, chemotherapy is also limited due to the toxic effects of the healthy cells, chemo resistance, and poor selectivity of anti-cancer drugs. Above all, BBB is the major limit for the delivery of chemotherapeutic agents that results in lower tumor accumulation of drug, tumor heterogeneity affecting sensitivity, and drug resistance [62]. Thus, novel strategies to further improve the brain tumor diagnostics and therapeutics is highly desired. Over the advantages of nanotechnology, several drug molecules were successfully encapsulated into the nanocarrier systems and deliver to brain or facilitate penetration through the BBB, thereby overcoming the previous drug delivery chemotherapeutic issues to unreachable tumors, such as glioblastoma multiforme (GBM) [63]. Subsequently, several kinds of nano-formulations were developed to load and deliver the hydrophilic and hydrophobic factors to the tumor site by crossing the BBB. For instance, prolonged half-life of Temozolomide (TMZ) was achieved around 13.4 h when it was encapsulated in the Chitosan-based NPs whereas a free drug having only a 1.8-h half-life [64]. Drug-loaded albumin NPs were recently found to target SPARC (secreted protein acidic and rich in cysteine) and gp60 (glycoprotein 60), which are overexpressed in glioma and tumor vessel endothelia [65]. Therefore, such pathways have been explored for use in brain-targeting biomimetic delivery. The albumin NPs also displayed enhanced BBB penetration, intra tumoral infiltration, and cellular uptake [66]. Overall, NPs exhibit great potential in preclinical studies. Besides the passive targeting strategy, active targeting might be employed to further promote the accumulation of therapeutic drugs at the brain tumor site. Another rat brain model examined the encapsulation of methotrexate-transferrin complexes and coating of polysorbate 80 on poly-lactic-co-glycolic acid NPs, finding better BBB-penetration, lower organ toxicity, and higher anti-tumor activity as compared with non-targeting NPs [67]. There is an upgraded need to further improve the compound solubility, stability, and reduce systematic toxicities of NPs.

**Figure 3:**
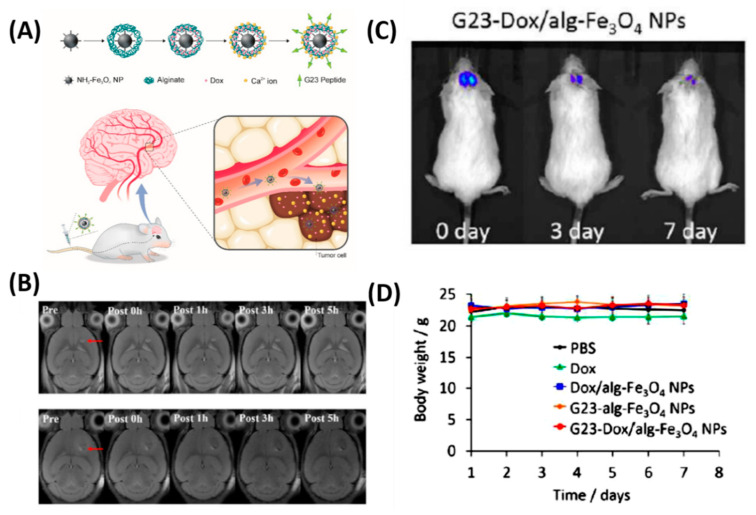
In vivo anti-tumor activity of G23-Dox/alg-Fe_3_O_4_ NPs. (**A**) Schematic representation of synthesis process and BBB penetrating Dox delivery. (**B**) In vivo MRI contrast imaging abilities of alg-Fe_3_O_4_ NPs and G23-alg-Fe_3_O_4_ NPs. (**C**) In vivo luminescence images show from U87MG-luc2 cells monitored using the IVIS imaging system after mice were intravenously injected with G23-Dox/alg-Fe_3_O_4_ NPs for seven days. (**D**) Body weights of mice during the treatment. Reproduced with permission from Reference [69].

Besides the chemo delivery platform, diagnostic tools such as a high-resolution imaging system before surgery is highly important for GBM, which are characteristically invasive. For instance, gadolinium NPs used as a magnetic resonance (MR) contrast agent can penetrate the BBB and are taken up by the brain tumor parenchyma [68]. To further achieve the therapeutic efficiency, diagnostic and chemotherapeutic platforms are attracted to monitor the tumor, especially for brain tumors. Thus, Cheng et al. synthesized a doxorubicin (DOX) nanocarrier composed of Fe_3_O_4_ NPs (particle size: 140 nm, zeta potential: −15 mV) and alginate, tagged with the BBB-permeating G23 peptides on the particle surface (G23-Dox/alg-Fe_3_O_4_ NPs) [69]. Tumors (U87MG) significantly shrank (from ~50 mm^3^ to a few mm^3^) in mice treated with G23-Dox/alg-Fe_3_O_4_ NPs after being intravenously injected with NPs for five days, which was confirmed by contrast-enhanced T2-weighted MRI images (Figure 3). In another study by Ni et al., it was demonstrated that the ANG/PEG-UCNPs nanoprobes targeted the glioblastoma efficiently via receptor mediated transcytosis [22]. Moreover, these nanoprobes greatly offer a MR imaging and near-infrared to near-infrared (NIR-to-NIR) upconversion luminescence (UCL) fluorescence imaging to visualize the tumors, which exhibited excellent performance that the clinically used MRI contrasts.

**Figure 4:**
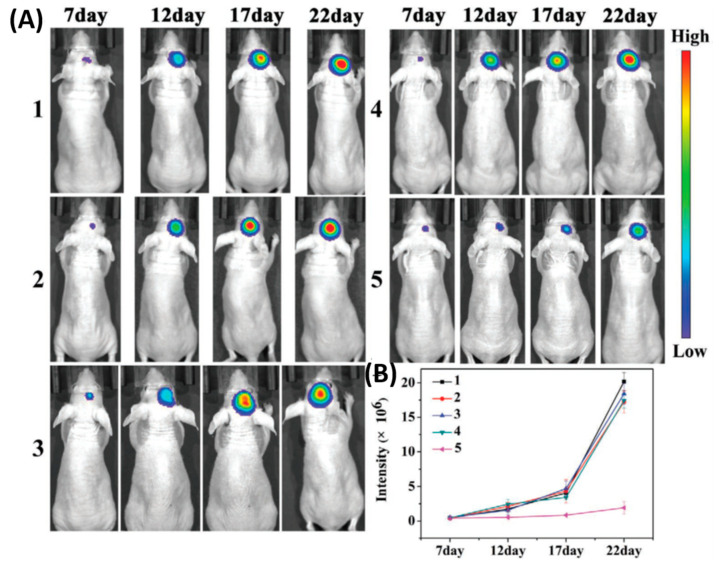
Cell membranes coated on ICG loaded nanoparticle (PCL-ICG) nanoparticles (NPs). (**A**) Representative bioluminescence images of U87MG-Luc glioma-bearing mice in different groups: (1) phosphate buffered saline (PBS), (2) normal cell coated ICG loaded nanoparticle (COS7-PCL-ICG), (3) COS7-PCL-ICG + laser, (4) B16-PCL-ICG, and (5) B16-PCL-ICG + laser under 808-nm laser irradiation (1 W cm^−2^, 5 min). CICG = 1 mg kg^−1^. (**B**) Quantitative fluorescence signal intensity in the brain. Reproduced with permission from Reference [74].

Thereafter, photo therapeutic approaches, such as photothermal therapy (PTT) and photodynamic therapy (PDT) to treat the brain tumor, gain significant attention. Due to the uneven light distribution and tumor hypoxia conditions, photo therapy alone could not kill the cancer cells efficiently and it is easy to induce the local reoccurrences and metastasis, especially for glioblastoma [70]. Hence, a combination of phototherapy with chemotherapy was highlighted to conquer the glioma tumor and metathesis. For instance, Liu’s group utilized the photosensitizer chlorin e6 (Ce6) conjugated with the anticancer drug paclitaxel (PTX) loaded human serum albumin (HSA) [71]. Furthermore, an acyclic Arg-Gly-Asp (cRGDyK) peptide conjugated to target the ανβ3-integrin, which was overexpressed on tumor angiogenic endothelia (HSA-Ce6-PTX-RGD). As a result, 2.4 times higher accumulation of targeted NPs was observed at the tumor site than with the bare NPs in vivo. The combination therapy allowed a prolonged survival rate of around 40 days whereas control groups exhibited 15–30 days. In another study, Yang Hu et al. reported the successful combination of phototherapy and gene therapy on polycationic Au NR-coated Fe_3_O_4_ nanospheres (Au@PDM/Fe_3_O_4_) to treat the C6 glioma tumor model [72]. The tumor suppressor gene P53 was loaded into the Au@PDM/Fe_3_O_4_ NPs, which significantly inhibits the tumor under 808-nm laser irradiation, after treatment volume of the tumor was reduced to 82% when compared to the control group. In another study, Hao et al. reported a tumor-targeting core-shell structured DTX-loaded PLGA@Au nanoparticles for image guided chemo-photothermal therapy to treat the GBM model [73]. NPs facilitate the improved delivery of chemo drugs and Au NPs help to track the accumulated NPs’ in vivo model. Furthermore, successful photothermal therapy achieved on Au NPs by exposing the 808-nm laser, present combination therapy that significantly improved therapeutic efficiency. Very recently, Wang et al. fabricated the cancer cell membrane camouflaged ICG-loaded polymeric nanoparticles (B16-PCL-ICG or 4T1-PCL-ICG) was constructed for treating early brain tumors via imaging and photothermal therapy [74]. As shown in Figure 4, the cell membrane camouflaged NPs exhibited a significantly higher accumulation at the tumor site by crossing the BBB than bare NPs, which was confirmed by stronger fluorescence signals that were observed in the brain of mice at 8 h. B16-PCL-ICG NPs could efficiently inhibit the glioma tumor growth under 808-nm laser irradiation mediated via PTT.

Over the advancement of nanotechnology, cell-membrane-coating NPs has attracted significant attention to construct as biomimetic drug delivery/therapeutic carriers [75]. These biomimetic nanocomposites can facilitate the mimicking of the tumor microenvironment that results in a wide range of favorable applications, such as specific targeting and prolonged circulation time. Although utilizing This strategy can treat cancer tumor models by using some cell membrane-coated nanostructures, [76,77] but, to use this strategy to treat brain tumors is rarely explored [78]. Over the years, there are numerous NPs that were successfully applied for brain tumors, which are summarized in Table 3.

**Figure 5:**
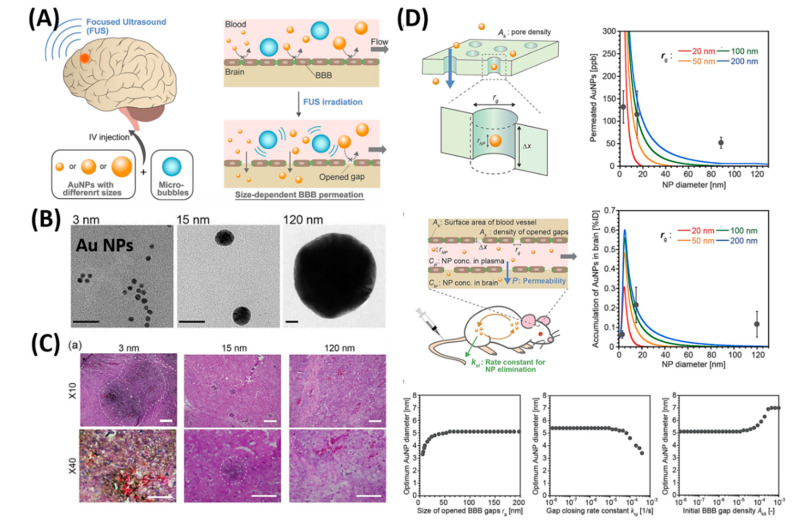
Focused ultrasound-induced blood–brain barrier opening strategy. (**A**) Schematic representation of size-dependent nanoparticle (NP) delivery to the brain via a focused ultrasound (FUS). (**B**) TEM images of 3, 15, 120-nm sized Au NPs. (**C**) Distribution of Au NPs in mouse brains in vivo models. (**D**) Kinetic modeling studies of FUS-assisted NPs delivery into the brain. Reproduced with permission from Reference [104].

Although NFs offer good beneficial advantages, restriction of their permeation through the BBB is even more pronounced due to the larger sizes of NPs. To this end, temporarily open the BBB techniques that are attracted in which a focused ultrasound (FUS) has an advantage capable of achieving non-invasive and targeted BBB disruption to promote the gene or drug delivery to the CNS [94]. As a result, several molecular drugs [95,96], antibodies [97,98], and oligonucleotides [99,100] were successfully delivered to the brain’s in vivo models via the FUS strategy. Although there are limited reports on NPs’ delivery via the FUS strategy, this strategy could provide a promising platform to deliver the NPs to the brain and mediate the therapeutics [101,102,103]. As shown in Figure 5, Ohta et al. investigated the size dependent delivery of Au NPs (30 to 120 nm) into the brain by crossing the BBB assisted by FUS [104]. In vivo experimental results reveal that smaller particles were not necessarily better for delivery systems, but the medium-sized Au NPs (15 nm) showed the highest delivery into the brain (2.2% ID via 0.7 MPa FUS) when compared to the smaller size (3 nm) and larger size of Au NPs (120 nm). The probable reason behind the size dependent permeability is mainly due to the competition between the permeation through BBB and excretion of particles from blood circulation. Experimental results exhibited that smaller NPs are preferable to deliver into the brain via BBB, but are quickly removed from the blood stream via kidneys. Besides, nose-to-brain delivery via intranasal administration of nano-formulations offers significant advantages such as easy penetration through the BBB and rapidly deliver the therapeutic drugs for the treatment of CNS disorders [105,106].

### 3.2. BBB-Penetrating NPs for Alzheimer’s Disease (AD)

**Figure 6:**
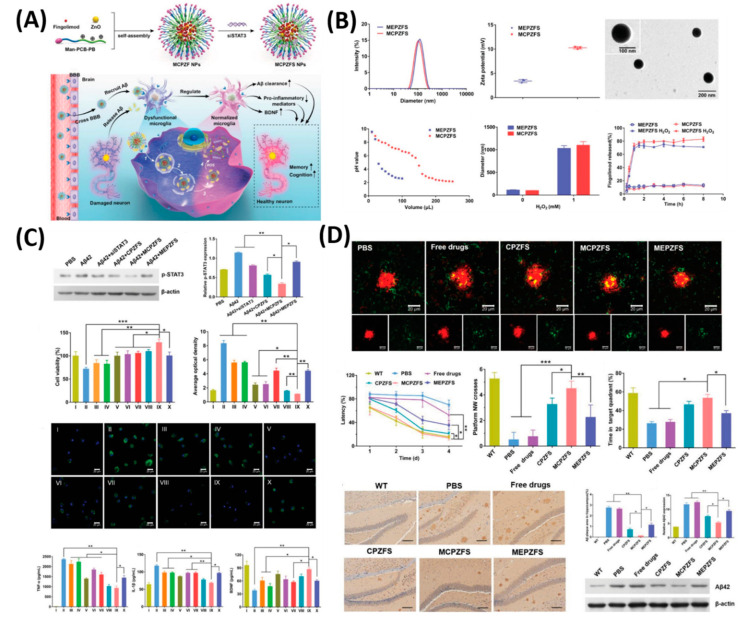
Zwitterionic poly(carboxybetaine) (PCB)-based nanoparticle (MCPZFS NPs) for treating Alzheimer’s disease (AD). (**A**) Schematic illustration of the MCPZFS NPs for AD. (**B**) Characterization of the NPs. (**C**) Effect of NPs on the inflammatory regulation of microglia and (**D**) the effect of NPs on phagocytosis and degradation of Aβ by microglia. Data are presented as the mean ± SD. * *p* < 0.05, ** *p* < 0.01, *** *p* < 0.001. Reproduced with permission from Reference [117].

AD is a chronic and progressive neurodegenerative disorder, which is a major factor in the onset of dementia and affects more than 5 million people in the US alone [107]. Memory loss is the main characteristic of AD and the greatest risk factor for AD is age. Spontaneous self-aggregation of Aβ plays an acute role in the etiology of AD [108]. Aβ aggregates may be responsible for triggering the neurotoxicity by inducing oxidative stress and inflammation responses in the brain of AD patients, leading to a cognitive defect and memory loss [109]. Therefore, it is very clear that either decreasing the Aβ production or inhibiting oxidative stress and inflammation in brains are the key therapeutic strategies for treating AD. Many studies of human AD patients and AD animal models suggest that cerebrovascular alterations result from the accumulation of the Aβ peptide [108,110]. Even though the presence of the Food and Drug Administration (FDA) approved available drugs to treat AD such as tacrine, donepezil, rivastigmine, galantamine, and memantine, an ideal carrier is highly desired to deliver the drugs to the brain via the BBB and increase the water solubility, in vivo half-life, and bioavailability [107,111]. It is proven that nanotechnology can deliver the drugs to the brain efficiently by crossing the BBB. Therefore, Liu et al. synthesized the B6 peptide (a transferrin substitute)-modified PEG-PLA NPs (B6-PEG-PLA NPs) and then loaded a neuroprotective peptide NAPVSIPQ (NAP) into B6-PEG-PLA NPs (B6-NAP/PEG-PLA NPs). NAPs could be released from B6-NAP/PEG-PLA NPs and accumulated in mice brains more effectively than when using NPs without B6 [112]. B6-NAP/PEG-PLA NPs and NPs without B6 were found to accumulate in the liver, lung, and spleen tissue. However, NPs without B6 were found to produce higher levels of tissue accumulation. In another study, NGF (nerve growth factor) was combined with NPs to treat AD disease because NGF is vital for central cholinergic neuron survival in the basal forebrain. NGF adsorbed on poly(n-butylcyanoacrylate) nanoparticles (PBCA) NPs coated with polysorbate-80 was administered in C57BL/6 mice and was found to accumulate significantly in the brain parenchyma 45 min after administration [113]. Radio-labeled ^125^I-clioquinol (CQ, an amyloid affinity drug) encapsulated polymeric n-butyl-2-cyanoacrylate (BCA) NPs (^125^I-CQ-PBCA NPs) was used for AD diagnosis [114]. In vitro and in vivo tests showed these NPs had high degrees of affinity for Aβ plaques. The ^125^I-CQ-PBCA NP brain uptake and retention in AD mouse brain was higher than that in free ^125^I-CQ-treated AD mice at 90 min after administration. Similar to B6-NAP/PEG-PLA NPs, Yin el al. synthetized sialic acid (SA)-modified selenium (Se) NPs conjugated with B6 peptides (B6-SA-Se NPs) [115], which shows high permeability across the BBB. B6-SA-Se NPs could effectively inhibit Aβ aggregation and disaggregate preformed Aβ fibrils into non-toxic small oligomers. Subsequently, various drug loaded poly(lactide-co-glycolide) nanoparticles (PLGA NPs) as a carrier, which was approved by FDA were reported to reverse cognitive deficits in an AD transgenic mouse model [116]. Another crucial strategy to accelerate the progression of AD is the vicious circle between and amyloid-β (Aβ) and dysfunctional microglia. To normalize the Aβ) and dysfunctional microglia establishment, Liu et al. reported a zwitterion poly(carboxybetaine) (PCB)-based nanoparticle (MCPZFS NP) system for an effective treatment for AD [117]. As shown in Figure 6, as a proof-of-concept, 84 nm of MCPZF NPs was synthesized and it offers superior siRNA condensation, which was evidenced by gel-electrophoresis. Furthermore, modification of PCB could efficiently facilitate endosomal/lysosomal escape by protonation and perturbation. Thereafter, we studied the effect of present NPs on the inflammatory regulation of microglia by essaying the p-STAT3 protein levels and levels of pro-inflammatory cytokines. Results exhibited that the MCPZFS NPs could significantly inhibit the Aβ-induced cytotoxicity by increasing the production of Brain-derived neurotrophic factor (BDNF) and decreasing the levels of proinflammatory cytokines, which might be attributed due to the excellent properties of NPs by escaping the endosomal/lysosomal. Besides, intracellular distribution of Aβ and NPs in BV2 cells further proved the enhanced microglial phagocytosis on the present system. On the other hand, small interfering RNAs (siRNAs) show a promising platform to treat the AD by silencing BACE1. However, a lack of carrier systems to deliver the siRNA to the brain is limited. Thus, Zhou et al. very recently reported glycosylated “triple-interaction” stabilized polymeric siRNA nanomedicine (Gal-NP@siRNA) to target BACE1 in a transgenic AD mouse model [118]. The results show the partial knockdown of BACE1 protein expression on the present NFs without noticeable side effects. These strategies indicated that Gal-NP@siRNA NFs has an excellent clinical translation potential for AD treatment owing to its stability, ease formulation, and successful BBB penetration.

### 3.3. BBB-Penetrating NPs for Parkinson’s Disease (PD)

PD is a progressive disease of the nervous system that affects a person’s movement, including writing and speaking. While the cause of the illness is still unknown, it is related to insufficient dopamine production by nerve cells in the brain. Currently, the most widely used strategy for PD treatment is dopamine replacement to improve motor function. To increase the dopamine concentration in the brain, direct dopamine infusion into the brain of PD animal models were reported but it has an unsuccessful end due to the fact that dopamine is not able to cross the BBB and, therefore, direct infusion is not possible, which results in behavioral abnormalities observed in animal models [119,120]. As a beneficial advantage of NPs, several nano carriers were developed to deliver the drugs to treat the PD efficiently. For instance, Huang et al. developed a neurotrophic factor gene (hGDNF, a plasmid for the human glial cell line)-loaded Polyamidoamine (PAMAM) and polyethyleneglycol (PEG) NPs modified by lactoferrin [121]. Glial cell line-derived neurotrophic factor (GDNF) is the golden standard neurotrophic factor for PD therapy. However, it is unable to cross the BBB. Lactoferrin-conjugated PAMAM and PEG NPs could deliver GDNF across the BBB to exert a neuroprotective effect on dopaminergic neurons. Thereafter, to deliver the dopamine to the brain efficiently via BBB, Pahuja et al. developed dopamine-loaded PLGA NPs (DA-PLGA NPs) that crossed the BBB mainly in the substantia nigra and striatum (PD-altered regions) of 6-hydroxydopamine rats [122]. In their study, DA-PLGA NPs prevent toxicity from bulk dopamine and provides a novel strategy to treat PD. Thereafter, other drugs like the ropinirole (RP) drug loaded into the PLGA NPs, were developed to demonstrate the drug delivery to the brain for treating PD with significant outcomes [123].

**Figure 7:**
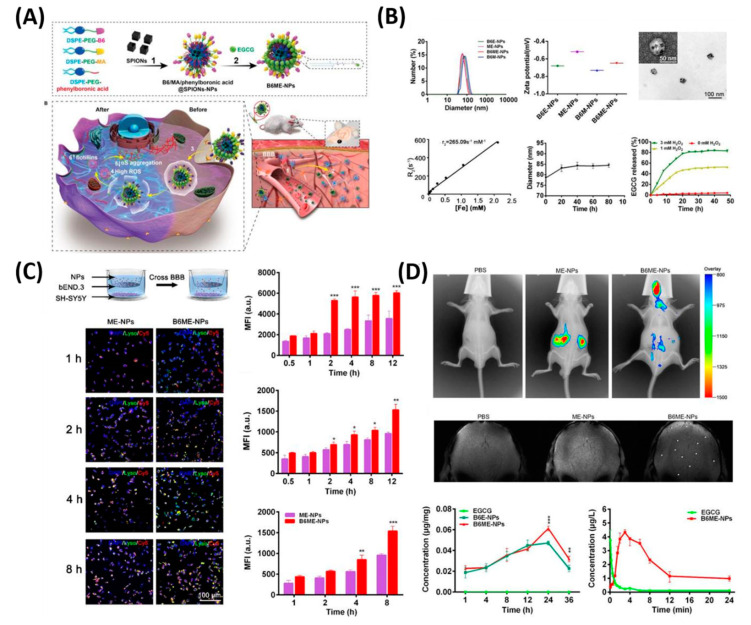
Dual-target traceable nanodrug for Parkinson’s disease (PD) treatment. (**A**) Schematic representation of synthesis of nanodrug and application for PD. (**B**) Systematic characterization of dual-target traceable nanodrug (B6ME-NPs). (**C**) Confocal microscopy (CSLM) and flow cytometry uptake studies to confirm the successful blood brain barrier (BBB) crossing. (**D**) Fluorescence and magnetic resonance (MR) images of the mice model after 24 h of i.v. injection of the nanodrug. Data are presented as the mean ± SD. * *p* < 0.05, ** *p* < 0.01, *** *p* < 0.001. Reproduced with permission from Reference [124].

In another feature of PD pathogenesis is α-synclein (αS) aggregation. This αS Aggregation could be prevented by Epigallocatechin gallate (EGCG). However, it is very difficult to accumulate the EGCG in vivo models to the BBB. Therefore, Li et al. reported cell-addictive,” traceable, ROS-responsive NPs with dual targets for delivering an Epigallocatechin gallate (EGCG) in dopaminergic neurons for treating PD [124]. As shown in Figure 7, the amount of EGCG accumulation in PD lesions was significantly enhanced on the fabricated B6ME-NPs. Moreover, incorporated superparamagnetic iron oxide nanocubes (SPIONs) helps to trace the drug molecules via magnetic resonance imaging. Finally, released EGCG inhibits αS aggregation and reduces the toxicity of dopaminergic neurons. Table 4 summarizes the various kinds of BBB penetrating therapeutic NFs for AD, PD, and stroke applications.

### 3.4. BBB-Penetrating NPs for Stroke

**Figure 8:**
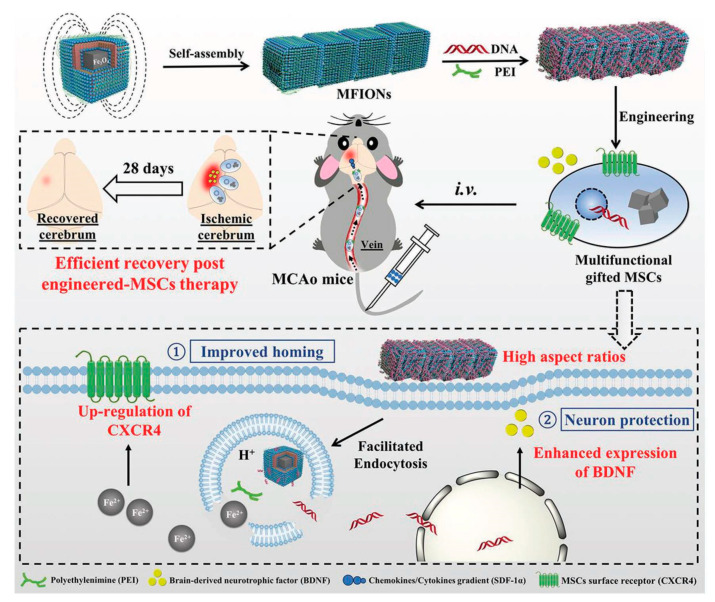
Schematic representation of magnetosome-like ferrimagnetic iron oxide nanochains (MFION)-based fabrication of Mesenchymal stem cells (MSCs) for the recovery of post-ischemic stroke. Reproduced with permission from Reference [151].

Stroke occurs when the blood supply to the brain is blocked. It can occur without warning and requires immediate medical attention. Globally, nearly 800,000 people have a stroke each year [139,140]. Lack of blood deprives brain cells of oxygen or nutrients, causing them to die, potentially causing severe damage to functions such as memory and muscle control. NPs can be used to deliver neuroprotective drugs to treat stroke-induced neuronal tissue damage. However, in their free form, neuroprotective drugs can only pass the BBB in very low amounts, and are quickly cleared by the reticuloendothelial system [141]. For example, specific caspase-3 inhibitor (Z-DEVD-FMK)-loaded chitosan NPs conjugated with a transferrin receptor antibody showed promising results for stroke treatment [137]. The nanocomposites were able to cross the BBB and decreased infarction volume (by about 40%) and neurological deficits caused by ischemia in a MCAO (middle cerebral artery occlusion) mice model of stroke. The nanocomposites were also able to repress caspase-3 activity. In addition to Z-DEVD-FMK, Tanshinone IIA, a phenanthrene-quinone derivative, has been proposed to induce neuroprotection and neuro-regeneration. Tanshinone IIA is a promising drug for treatment of oxidative stress in neurological disorders [142]. However, Tanshinone IIA has a short half-life in circulation, poor water-solubility, and low BBB penetration [143]. To overcome these problems, bovine serum albumin-conjugated tanshinone IIA PEG NPs were developed and used for a Middle cerebral artery occlusion (MCAO) rat model. This nanocomposite could decrease infarction volume by approximately 70%, reducing the neurological deficit and neuronal apoptosis in an MCAO rat treated with NPs [144]. Adenosine is another molecule with significant potential for neuroprotection [145]. However, adenosine has moderate toxicity and a short half-life in circulation. NPs formulated by the conjugation of adenosine with squalene were developed to resolve both problems [146]. The functionalized NPs decreased the infarction area and enhanced neurological deficit scores. Subsequently, various delivery and therapeutic NFs, such as T7 peptide and stroke homing peptide (SHp, CLEVSRKNC)-conjugated liposome (T7&SHp-P-LPs/ZL006) [147], liposomal formulation [148], SHp-RBC-NPs [149], melanin [150], and Edaravone-Loaded Ceria nanoparticles [135] have been designed to achieve efficient treatments for stroke applications with significant outcomes. Mesenchymal stem cells (MSC) based on therapeutic approaches pay significant attention by its potential benefits but, due to the insufficient delivery to damaged tissues and insufficient secretion of neuroprotective factors, makes them limited for practical applications so far. To overcome these issues, Zhang et al. demonstrated a non-viral, magnetic field-independent gene transfection approach by using a magnetosome-like ferrimagnetic iron oxide nanochains (MFIONs) to treat the post-stroke recovery [151]. As shown in Figure 8, the present platform offers a favorable cellular uptake and high stem cell gene delivery. Moreover, ferrous ions released from MFIONs can efficiently excite the upregulations of CXCRC4. Finally, high r2 relaxivity of MFIONS allow sensitive and non-invasive monitoring of MRI. However, most of the NP strategies suffer from shorter vascular circulation time, aggregation, and other undesirable catalytic reaction at active sites, which makes them limited for further clinical development. Recently, He et al. developed a bioactive zeolitic imidazolate framework-8–capped ceria nanoparticles (CeO_2_@ZIF-8 NPs) for improving the therapeutic efficiency of ischemic stroke [152]. The present nanoplatform offers improved BBB permeation, prolonged blood circulation times, and more accumulation in the brain makes them potential agents to inhibit the lipid peroxidation in the brain tissues and reduces the oxidative damage and apoptosis of neurons in the brain tissue. It also suppresses the inflammation and immune response-induced injuries by suppressing the activation of astrocytes and secretion of proinflammatory cytokines, thus, achieving satisfactory prevention and treatment in neuroprotective therapy. As known, the adhesion of neutrophils to endothelial cells triggers the initiation of inflammation in ischemia/reperfusion (I/R). Based on this concept, Dong et al., developed neutrophil membrane-coated NFs loaded with a Resollvin D2 (RvD2) drug to prevent neuroinflammation. This platform offers an enhancing resolution of inflammation during ischemic stroke therapy [133].

## 4. Conclusions and Future Perspectives

The BBB forms a natural shield, which prevents therapeutic drugs (i.e., traditional methods) as well as NP-based therapeutic platforms (i.e., nanotechnology-based methods) from accessing brain tissue. Some strategies have been developed to enhance the transport of NP formulations across the BBB, such as the use of certain ligands on the NP surface. These ligands include peptides, antibodies, and proteins and help NPs to cross the BBB though receptor-mediated pathways. Ligand-conjugated NPs could encapsulate anti-cancer or neuroprotective drugs for targeted therapy. Notably, results were proven that the use of NPs could enhance local drug concentrations, thus, reducing overall drug dosage required and associated side effects. However, available clinical trial data are limited, but current studies suggest that NPs have great potential in the detection, diagnosis, and therapy of brain-related diseases. Some important issues should be considered in future applications such as (i) long-term health effects of NPs that are currently unknown and require further study, along with their biodistribution, side effects, pharmacokinetics, toxicity, and role in therapeutic strategies. (ii) Size, charge, and shape of NPs are greatly effecting the BBB penetration, need to find the optimal particle size, (iii) protection of theranostic platform for successful macrophage escape is highly important to enhance the therapeutic efficiency, and (iv) selective targeting to the brain by using brain targeting ligands will be a key role to minimize the side effects to major organs. (v) Successful conjugation of brain targeting ligands with image-guided tracking agents for real-time monitoring the therapeutic effects will be a promising strategy. (vi) More efforts should be devoted to develop combined therapeutic strategies, which include a combination of two or three functional properties on a single NF, including PTT, PDT, chemotherapy, immunotherapy, radiotherapy, gene therapy, and magneto thermal therapy to further improve the therapeutic efficacy against glioblastoma. (vii) Fabricating the NPs with cell-penetrating peptides will be a potential candidate to avoid the endocytotic pathway for successful delivery of NPs into the cell cytoplasm. (viii) Moreover, NPs/NFs should be cost-effective and the physical properties of NPs/NFs should be manipulated easily, according to the mode of delivery. Overall, we strongly envision that the present review will drag more scientific attention to understand the principles of BBB-overexpressed receptors and fabricate an efficient BBB-penetrating NFs for conquering the brain related to disorders in future applications.

## Figures and Tables

**Table 1:** Key strategies to overcome the current brain-related disease treatments by nanotechnology.

Treatment Strategies	Problems	Troubleshoot with Nanotechnology
Surgery	Difficult to identify the tumor boundaries	Intraoperative [25] and near infrared fluorescence (NIRF) imaging [26] based on nanoparticle (NP) probes to differentiate the clear tumor margins
Radiotherapy	Radio resistance	○ Deliver nanoparticle-based radio sensitizer [27]. ○ Deliver O2-generating NPs to alleviate tumor hypoxia [28]. ○ Deliver gases such as NO and H2S, releasing nanoplatforms (NFs) [29]. ○ Photo-radio combined NFs [30]
Chemotherapy	○ BBB	○ Targeting-drug loaded NFs for blood brain barrier (BBB) crossing and active tumor binding [31].
	○ Low accumulation of drug	○ Encapsulating the drugs into nanocarriers to improve the accumulation in the brain [32].
	○ Tumor heterogeneity affecting sensitivity	○ Cell penetrating peptides for deep tumor therapeutics [33]
	○ Drug resistance	○ “all-in-one” NFs for combinational therapy [34]

**Table 2:** Some receptors and transporters overexpressed on the blood brain barrier (BBB).

Receptor Mediated Transport	Active Flux Mediated Transport	Transporter Mediated Transport
Transferrin receptor [43], Nicotinic acetylcholine receptor [44], Insulin receptor [45], Leptin receptor [45], Lipoprotein receptor [46], Neonatal Fc receptor [38], Diphtheria toxin receptor [45]	Taurine transporter [37], Amino acid transporter [47], Polypeptide transporter [37], Organic anion transporter [45], ATP-binding cassette (ABC) transporter, P-glycoprotein [48]	Nucleobase transporter [37], Glucose transporter [49], Cationic amino acid transporter [45], Choline transporter [50], Mono carboxylic transporter [45], Large neutral amino acid transporter [37]

**Table 3:** Literature summary of BBB penetrating nanoparticles (NPs) to mediate the brain tumor therapeutics.

S. No.	Nanoplatforms (NF)	Target Ligand	Therapeutic Features	Ref.
1	Fe_3_O_4_ NPs	Lactoferrin	Imaging	[51]
2	Polymersome	G23 peptide	Drug Carrier	[52]
3	RGD-QDs	RGD peptide	NI Imaging	[79]
4	EGFpep-Au NPs	EGF peptide	PDT	[80]
5	G4-DOX-PEG-Tf-TAM	Transferrin (Tf)	Drug delivery	[81]
6	ANG-PEG-NP	Angiopep-2	Drug delivery	[46]
7	PBCA NPs	Polysorbate 80	Delivery	[82]
8	DTX-ANG20/TAT10-Ms	Angiopep-2	Imaging, drug delivery	[83]
9	ANG-IMNPs	angiopep-2	PTT/PDT	[84]
10	Tw-Mtx-Tf-NP	Transferrin	Drug delivery	[67]
11	AP-PLGA-NPs	Polysorbate	Drug delivery	[85]
12	TAT-Au NP	TAT peptide	Drug delivery, MR imaging	[54]
13	DOX-EDT-IONPs	Passive	Chemotherapy	[86]
14	(ICG)-loaded polymeric NPs	Passive	Imaging, PTT	[74]
15	^131^I-Au PENPs-CTX	Chlorotoxin	Imaging, Radio therapy	[87]
16	MoS2–ICG NSs	Passive	PA Imaging	[88]
17	mPEG-PLGA NPs	Passive	Dual drug delivery	[89]
18	LP-iDOPE	Passive	NIR imaging, Photo-immune therapy	[90]
19	Fe_3_O_4_ NPs	G23 peptide, passive	MR Imaging, drug delivery	[91,92]
20	B16-PCL-ICG NPs	Cell membrane	Fluorescence imaging, PTT	[74]
21	BLIPO-ICG NPs	Cell membrane	Fluorescence imaging, PTT	[93]

Abbreviations: arginine-glycine-aspartate (RGD), Quantum dots (QDs), epidermal growth factor peptide (EGFpep), Doxorubicin (DOX), Polyethylene glycol (PEG), Tamoxifen (TAM), Angiopep (ANG), poly(n-butylcyanoacrylate) NPs (PBCA NPs), docetaxel (DTX), transactivator of transcription (TAT), methotrexate (MTX), Acetylpuerarin (AP), poly(lactic-co-glycolic acid) (PLGA), trimethoxysilylpropyl-ethylenediamine triacetic acid (EDT), iron oxide nanoparticles (IONPs), Indocyanine green (ICG), chlorotoxin (CTX), polyethylenimine NPs (PENPs), Molybdenum sulfide (MOS_2_), liposomally formulated phospholipid-conjugated indocyanine green (LP-iDOPE), poly(ε-caprolactone)(PCL), liposome (BLIPO).

**Table 4:** Literature summary of the blood brain barrier (BBB) penetrating nanoparticles (NPs) for Alzheimer’s disease (AD), Parkinson’s disease (PD, and stroke applications.

S. No.	Nanoplatforms (NF)	Disease Model	Therapeutic Strategy	Ref.
1	HMON-abAβ40	AD	Aβ1-40 peptide, MR imaging	[125]
2	Liposome NPs	AD	Carrier, MR, and NIRF imaging	[126]
3	GSH-Au NPs	AD	inhibition of Aβ42	[127]
4	PEG–PLGA NPs	AD	Memantine delivery	[128]
5	B6-SA-Se NPs	AD	inhibition of Aβ42	[115]
6	MCPZFS NP	AD	inhibition of Aβ42	[117]
7	Gal-NP@siRNA	AD	silencing of BACE1	[118]
8	DP-PLGA NPs	PD	Dopamine delivery	[122]
9	PLGA NPs	PD	Ropinirole delivery	[123]
10	B6ME-NPs	PD	EGCG delivery, MR imaging	[124]
11	Tf-TMD-PLGA-NP	PD	Tramadol delivery	[129]
12	PS 80-modified-CPC	PD	curcumin nanocarrier	[130]
13	Lf-BP-Pae	PD	Paeoniflorin (Pae) delivery	[131]
14	Dex-IO NPs	PD	Improve the human MSCs (hMSCs)	[132]
15	RvD2-HVs	Stroke	Decrease TNF-α and alleviate inflammation responses	[133]
16	pSV-HO-1/R3V6-Dexa	Stroke	Dexamethasone drug delivery	[134]
17	E-A/P-CeO_2_	Stroke	ROS scavenging ability	[135]
18	Mn_3_O_4_@nanoerythrocyte-T7 (MNET)	Stroke	scavenged free radical and oxygen supply	[136]
19	Chitosan NPs	Stroke	basic fibroblast growth factor (bFGF) and a small peptide inhibitor of caspase-3	[137]
20	Protein-Carbon Dot Nanohybrid	Stroke	early detection of BBB damage and thrombolytic agent distribution	[138]

Abbreviations: hollow manganese oxide nanoparticles (HMON), antibody of Aβ1-40 peptide (abAβ40), glutathione (GSH), sialic acid (SA), selenium (Se), zwitterion poly(carboxybetaine) (PCB)-based nanoparticle (MCPZFS NP), Small interfering RNAs (siRNAs), dual-target traceable nanodrug (B6ME-NPs), Tramadol (TMD), curcumin (CPC), lactoferrin (Lf), Paeoniflorin (Pae), black phosphorus nanosheets (BP), Dextran (Dex), Resolvin D2 (RvD2), heme oxygenase-1 (HO-1), Dexamethasone (Dexa), R3V6 peptide (R3V6), Cerium dioxide (CeO_2_), Angiopep-2 and poly(ethylene glycol) (E-A/P), and manganese oxide (Mn_3_O_4_).
